# The Dedication of Meharry Dental and Pharmaceutical College

**Published:** 1889-12

**Authors:** 


### THE DEDICATION OF MEHARRY DENTAL AND PHARMACEUTICAL COLLEGE.

---

The new Dental and Pharmaceutical Building of Meharry Medical Department of Central Tennessee College, Nashville, Tenn., was dedicated November 20th. Addresses were delivered by W. H. Morgan, M. D., D. D. S., dean of the Dental Department of Vanderbilt University; ex-President Rutherford B. Hayes; ex-Post-Master General D. McKey; Rev. J. C. Hartzell, D. D., Corresponding Secretary of the Freedmen's Aid and Southern Education Society of the Methodist Episcopal Church; Rev. C. S. Smith, M. D., of the A. M. E. Sunday School Union, Nashville, Tenn.; and Dr. J. B. Lindsley, Secretary of the Tennessee State Board of Health.

The building is constructed of brick with the exception of the first story which is stone, it is sixty feet long and forty feet wide. The first story will contain a dental laboratory, and a chemical laboratory for analytical work; the second a dental infirmary, a waiting room and two rooms for pharmacy; the third and fourth stories are occupied by an amphitheater, capable of seating two hundred students, with a sky light over the place designed for the operating table; there is also a room for the accommodation of patients, a faculty room and a museum. The Dental Infirmary is thirty-seven feet long and twenty-four feet wide, wainscoted and paneled in oak, lighted with twelve large windows.

The School of Dentistry was organized for the education of colored students, and has been in operation three years, the graduates of which are doing good work, and have been well received by the dental profession. This school is a member of the "National Association of Dental Faculties," and its diplomas are recognized by the "National Association of Dental Examiners."

